# Diagnosis and phylogenetic analysis of Orf virus from goats in China: a case report

**DOI:** 10.1186/1743-422X-7-78

**Published:** 2010-04-25

**Authors:** Keshan Zhang, Zhongxin Lu, Youjun Shang, Haixue Zheng, Ye Jin, Jijun He, Xiangtao Liu

**Affiliations:** 1 Lanzhou Veterinary Research Institute of Chinese Academy of Agriculture Science, State Key Laboratory of Veterinary Etiological Biology, National Foot-and-Mouth Disease Reference Laboratory, Key Laboratory of Animal Virology of Ministry of Agriculture, Xujiaping No.1, Yanchangpu, Lanzhou, Gansu, 730046, China

## Abstract

**Background:**

Orf virus (ORFV) is the etiological agent of contagious pustular dermatitis and is the prototype of the genus Parapoxvirus (PPV). It causes a severe exanthematous dermatitis that afflicts domestic and wild small ruminants.

**Case presentation:**

In the present study, an outbreak of proliferative dermatitis in farmed goats. The presence of ORFV in tissue scrapings from the lips was confirmed by B2L gene polymerase chain reaction (PCR) amplification. The molecular characterization of the ORFV was performed using PCR amplification, DNA sequencing and phylogenetic analysis of the B2L gene.

**Conclusion:**

The results of this investigation indicated that the outbreak was caused by infection with an ORFV that was closely related genetically to Nantou (DQ934351), which was isolated from the Tai wan province of China and Hoping (EU935106), which originated from South Korea in 2008. This is the first report of the phylogenetic analysis of ORFV from goats in China.

## Background

The ORFV is the prototype member of the genus *Parapoxvirus*, which also includes *pseudocowpox viru*s (PCPV) in cattle, *bovine papular stomatitis virus *(BPSV) in cattle, *squirrel parapoxvirus *(SPPV) and *parapoxvirus of red deer in New Zealand *(PVNZ) [1,2]. Contagious pustular dermatitis is a common viral skin disease that occurs in a range of species, not only in wild ruminants [3] but also in humans [4-6]. Humans with immunodeficiency diseases, in particular, can develop serious infections [7]. The diseases caused by ORFV have worldwide distribution and have been reported from many countries [1].

The disease not only has an economic impact on farmers worldwide but also has a considerable negative effect on animal welfare. Infected animals are sickly, fail to thrive, and are more susceptible to adventitious bacterial infections [8]. The typical progress of orf in goats and sheep moves from erythema, via vesicle formation, to pustules and then to scabs. Characteristic of the disease are proliferative and often self-limiting lesions on the skin of the lips, on the oral mucosa and around the nostrils. Lesions can also be found occasionally on the teats of nursing animals and rarely on other organs[9]. Depending on the location of the lesions, animals may be unwilling to nurse, eat, or walk [10]. Primary lesions usually resolve spontaneously within 3-4 weeks [11]. The mortality rate related to orf is usually low, but it may be very high in small ruminants, especially when bacterial or fungal secondary infections occur [12,13]. The ORFV genome consists of linear double-stranded DNA (134-139 kb) [14]. The envelope gene (B2L) of the ORFV encodes for a highly immunogenic envelope protein of about 42 kDa [15]. A conventional PCR method that is based on the amplification of the B2L gene has been used for the detection of ORFV by PCR [1,16,17]. Molecular characterization and phylogenetic analysis [1,18,19] have been based on the complete sequence of the B2L gene.

In recent years, outbreaks of orf have occurred worldwide [1]. Although outbreaks of orf have occurred in China and have been confirmed, there are few reports available of the detailed molecular characteristics and phylogenetic analysis of the viruses involved. We report an outbreak of ORFV infection in goats from the Hubei province of the People's Republic of China in which the ORFV was verified by PCR of the full-length B2L gene. Comparative sequence analysis of the B2L gene from this outbreak of orf was carried out, and the phylogenetic relationship of the virus with other ORFV sequences available in GenBank was determined. This is the first report of the phylogenetic analysis of an ORFV in China in comparison with other isolates from other regions.

## Case presentation

The outbreak reported in this study originated on a goat farm (114.52° E, 29.6° N) in the Hubei province of the People's Republic of China. On 23 March 2009, the farmer bought 655 goats from free-ranging herds and transported them a distance of about 800 km to the farm. On the third day, two goats presented with nodular lesions on the lips, tongue and around the mouth; there were 30 goats showed tubercular lesions three days later. To 31 July 2009, the incidence was approaching 60% and the mortality rate was 24.7% (162/655), although anti-viral and antibiotic medicines were administered in the drinking water, by intramuscular injection or orally.

Two goats with typical clinical signs were selected and euthanized with an overdose of pentobarbital sodium. Complete post mortem examinations were performed and the macroscopic changes were recorded. Small samples of diseased tissue, about 6-8 mm^3^, were removed carefully from the nodular lesions using a sterile scalpel and stored in a sealed plastic tube with 50% glycerin Phosphate Buffered Saline(PBS) at 20°C until analysis. Primers designed for the amplification of the full length of the B2L gene [20,21] were synthesized (Takara dalian Co., Ltd). Tissue scrapings collected from the infected goats were triturated in 0.1 M PBS (1:10 V/V) and freeze-thawed twice between -20°C and 37°C, then placed at 4°C overnight. After centrifugation at 3000 rpm for 20 min at 4°C, DNA was isolated from the clarified supernatant using a Genomic DNA Purification Kit (Promega) and used as template in the PCR procedures. Tissue scrapings of healthy goat were treated with the same procedures and used as negative control.

Comparison of the B2L sequences with those available in the GenBank database was performed using the online BLAST programs. The sequence identities of nucleotides and amino acids were analyzed by the ClustalW method [22]. A phylogenetic tree based on the deduced amino acid sequences was constructed by the neighbor-joining method with 1000 bootstrap replicates using MEGA version 4.0 [23].

## Discussion and Conclusion

**Figure 1:**
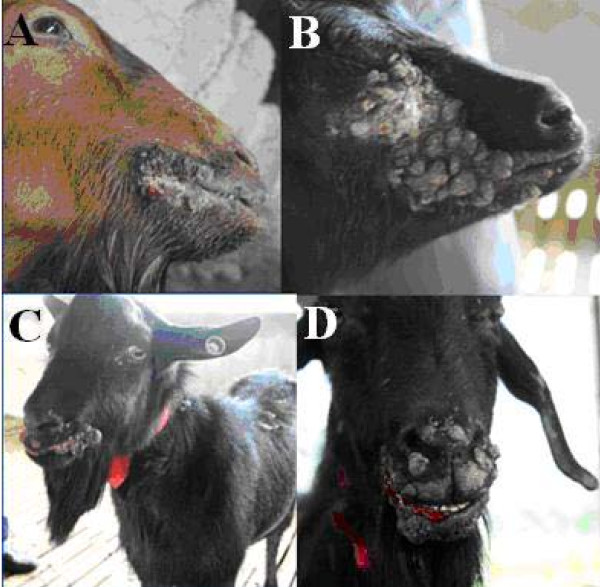
**Representative clinical cases of ORFV infection in goats**. (A) Goat with severe proliferative lesions of ecthyma around the lips. (B and C) Wart-like multiple nodules on the upper and lower eyelids. (D) Copious serosanguineous discharge appeared when the nodules burst.

The clinical signs seen in the goats included: multifocal to coalescing papillary, verrucose, proliferative, and ulcerated lesions in the epidermis of the muzzle and lips (Fig. 1). No visible lesions were found in other locations.

**Figure 2:**
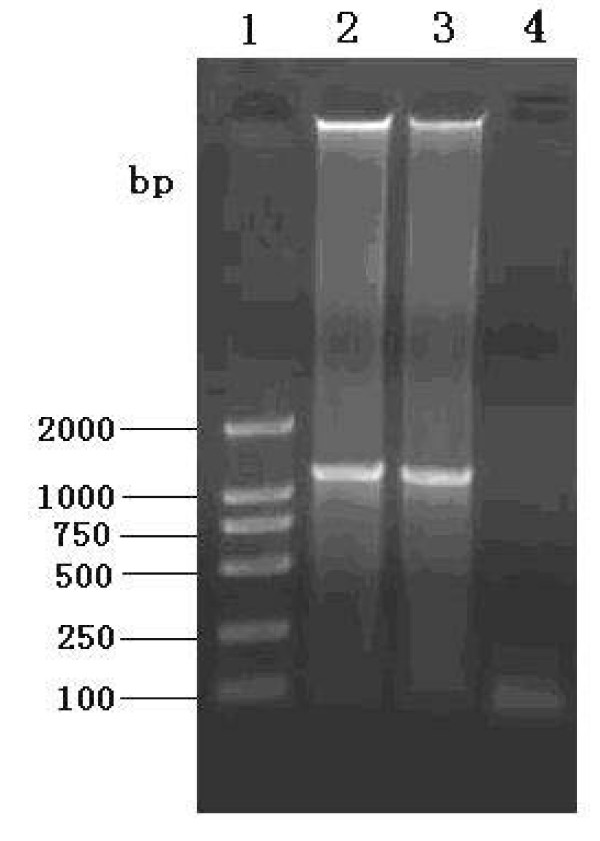
**Agarose (0.8%) gel electrophoresis of major envelope (B2L) gene fragment obtained by PCR, stained with ethidium bromide**. Lane 1: DNA ladder markers 2000 bp; Lanes 2 and 3: PCR products of complete B2L gene (1137 bp); Lane 4: negative control PCR using PBS as template DNA.

The orthodox methods of diagnosis that depend on pathologic examinations and clinical signs are inaccurate, virus isolation is thought to be a gold standard but it's time-consuming[14]. With the development of molecular biology, the PCR technique has become widely used to amplify the desired genomic fragments from tissue specimens, and it has become a powerful tool in molecular diagnosis. The PCR method is able to diagnose ORFV infection in field specimens from affected animals [2]. To confirm whether the causative agent was present in skin scrapings, PCR of the complete B2L gene was used in this study. The expected PCR fragments, approximately 1137 bp in length, were obtained from DNA which had been extracted from tissue scrapings; no fragments were obtained from the negative control (Fig. 2). To ensure the validity of the sequence as far as possible, DNA probest Taq enzyme was used in the PCR, and the PCR products were amplified directly from the skin scrapings. To gain further information about the virus, we sequenced the PCR product. The results showed that the B2L gene was 1137 bp in length and encoded 379 amino acids. The G+C ratio was 63.3%, which is consistent with the whole genome of the virus [1,24]. Sequences of the B2L gene of the virus were submitted to NCBI GenBank and assigned the accession number GU320351.

**Figure 3:**
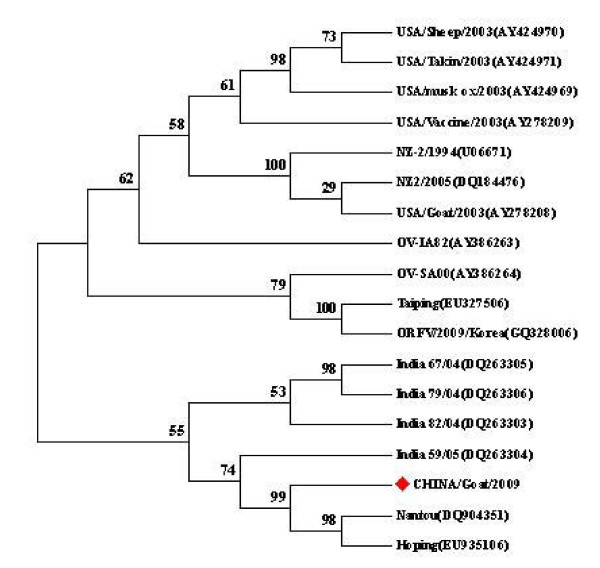
**Phylogenetic analysis based on deduced amino acid sequence of complete B2L gene**. The phylogenetic tree was constructed by the neighbor-joining algorithm using MEGA 4.0, and bootstrap analysis was performed with 1000 trials. All sequences were collected from GenBank. The red spot indicates CHINA ORFV, isolated in this study.

Using nucleotide sequences of the complete B2L gene stored in GenBank (Table 1), the phylogenetic relationships were explored using the neighbor-joining method and bootstrap analysis (Fig. 3). The results demonstrated that the CHINA/Goat/2009 isolate obtained from this outbreak was closest to the Nantou (DQ934351) isolate obtained from the Tai wan province of China in 2006 and the Hoping (EU935106) isolate, which originated from South Korea in 2008 (Fig. 3). The percent identities and diversities of the deduced amino acid sequence of the B2L gene among the different strains of ORFV were calculated (Table 2) using the MegAlign function of DNASTAR software. The sequence analysis revealed high nucleotide and amino acid identity among the isolates from different countries; they shared 95.3%-99.7% sequence identity at the amino acid level. This is consistent with the fact that the central region of Parapoxviruses is generally conserved.

**Table 1:** Detailed information on the ORFV used in the analysis; "---" indicates host species unknown.

**S.No**.	Virus strains	Country and year of isolation	Accession Number	Host species
1	NZ2/2005	New Zealand 2005	DQ184476	---
2	USA/Goat/2003	USA 2003	AY278208	Goat
3	USA/Vaccine/2003	USA 2003	AY278209	---
4	OV-IA82	USA 1982	AY386263	Lamb
5	OV-SA00	USA 2003	AY386264	Kid
6	USA/musk ox/2003	USA 2003	AY424969	Musk ox
7	USA/Sheep/2003	USA 2003	AY424970	Sheep
8	USA/Takin/2003	USA 2003	AY424971	Takin
9	India 82/04	India 2004	DQ263303	Goat
10	India 59/05	India 2005	DQ263304	Goat
11	India 67/04	India 2004	DQ263305	Sheep
12	India 79/04	India 2004	DQ263306	Sheep
13	Nantou	CHINA tw 2006	DQ904351	Goat
14	Taiping	CHINA tw 2007	EU327506	---
15	Hoping	South Korea 2008	EU935106	Goat
16	ORFV/2009/Korea	South Korea 2009	GQ328006	Dairy goat
17	NZ-2/1994	New Zealand1994	U06671	Sheep
18	CHINA/Goat/2009	China	GU320351	Goat

**Table 2:** The percentage identities and diversities of deduced amino acid sequences of the B2L gene among ORFV strains.

	1	2	3	4	5	6	7	8	9	10	11	12	13	14	15	16	17	18		Virus strains
**1**		99.5	98.7	97.6	96.3	97.4	99.5	97.1	97.1	97.6	97.6	98.4	98.2	96.8	97.1	97.4	96.6	96.8	**1**	CHINA Goat 2009
**2**	0.3		98.9	97.9	96.6	97.6	99.7	97.4	97.4	97.9	97.9	98.7	98.4	97.1	97.4	97.6	96.8	97.1	**2**	Hoping(EU935106)
**3**	1.1	0.8		98.2	96.8	97.4	98.9	97.6	97.6	97.6	98.2	98.4	98.2	97.4	97.6	97.9	97.1	97.4	**3**	India 59 05(DQ263304)
**4**	2.1	1.9	1.6		98.4	98.4	97.9	98.7	98.7	97.1	97.6	97.9	97.6	98.4	97.1	97.4	96.6	97.4	**4**	India 67 04(DQ263305)
**5**	3.5	3.2	3.0	1.3		97.1	96.6	97.4	97.4	95.8	96.3	96.6	96.3	97.1	95.8	96.0	95.3	96.0	**5**	India 79 04(DQ263306)
**6**	2.4	2.1	2.4	1.3	2.7		97.6	97.4	97.4	96.8	97.9	97.6	97.4	97.1	96.8	97.1	96.3	97.1	**6**	India 82 04(DQ263303)
**7**	0.3	0.0	0.8	1.9	3.2	2.1		97.4	97.4	97.9	97.9	98.7	98.4	97.1	97.4	97.6	96.8	97.1	**7**	Nantou (DQ904351)
**8**	2.7	2.4	2.1	1.1	2.4	2.4	2.4		99.7	97.1	98.2	97.9	97.6	99.5	98.2	98.4	97.6	98.4	**8**	NZ-2 1994(U06671)
**9**	2.7	2.4	2.1	1.1	2.4	2.4	2.4	0.0		97.1	98.2	97.9	97.6	99.5	98.2	98.4	97.6	98.4	**9**	NZ2 2005(DQ184476)
**10**	2.1	1.9	2.1	2.7	4.1	3.0	1.9	2.7	2.7		97.1	97.9	99.2	96.8	96.6	96.8	96.0	96.3	**10**	ORFV 2009Korea(GQ328006)
**11**	2.1	1.9	1.6	2.1	3.5	1.9	1.9	1.6	1.6	2.7		97.4	97.6	97.9	98.7	98.9	98.2	98.4	**11**	OV-IA82(AY386263)
**12**	1.6	1.3	1.6	2.1	3.5	2.4	1.3	2.1	2.1	2.1	2.7		98.4	97.6	97.4	97.6	96.8	97.1	**12**	OV-SA00(AY386264)
**13**	1.6	1.3	1.6	2.1	3.5	2.4	1.3	2.1	2.1	0.5	2.1	1.6		97.4	97.1	97.4	96.6	96.8	**13**	Taiping (EU327506)
**14**	3.0	2.7	2.4	1.3	2.7	2.7	2.7	0.3	0.3	3.0	1.9	2.4	2.4		97.9	98.2	97.4	98.2	**14**	USA Goat 2003(AY278208)
**15**	2.7	2.4	2.1	2.7	4.1	3.0	2.4	1.6	1.6	3.2	1.1	2.7	2.7	1.9		98.9	98.2	98.4	**15**	USA musk ox 2003(AY424969)
**16**	2.4	2.1	1.9	2.4	3.8	2.7	2.1	1.3	1.3	3.0	0.8	2.4	2.4	1.6	0.8		98.9	98.9	**16**	USA Sheep 2003(AY424970)
**17**	3.2	3.0	2.7	3.2	4.6	3.5	3.0	2.1	2.1	3.8	1.6	3.2	3.2	2.4	1.6	0.8		98.2	**17**	USA Takin 2003(AY424971)
**18**	3.0	2.7	2.4	2.4	3.8	2.7	2.7	1.3	1.3	3.5	1.3	3.0	3.0	1.6	1.3	0.8	1.6		**18**	USA Vaccine 2003(AY278209)

	**1**	**2**	**3**	**4**	**5**	**6**	**7**	**8**	**9**	**10**	**11**	**12**	**13**	**14**	**15**	**16**	**17**	**18**		

Orf is endemic in China, although a vaccination program has been performed to control the disease. Between 1980s and 1990s, orf occurred in eight provinces of China including Qinghai, Gansu, Tibet, Xinjiang, Liaoning, Jiangxi, Heilongjiang and Hebei. In recent years orf happened in the following provinces Innermongolia (Jun.2005), Guangxi (Mar.2005), Shanxi (Apr.2005), Fujian (Aug.2005), Jilin (Mar.2006), Jiangsu(Nov.2006) and Beijing city (Apr.2006). More seriously, in 2005 there were seven women and four men were infected by ORFV in Fujian province. So, orf is a national zoonoses in China. In this study, we diagnosed an outbreak of orf in Chinese goats and determined its phylogenetic characteristics on the basis of the complete gene sequence of the major envelope protein (B2L). We conclude that the Chinese ORFV involved in this outbreak was closely related phylogenetically to Nantou (DQ934351) and Hoping (EU935106). This is the first report to provide phylogenetic information about an ORFV strain in China, which will be of use for prospective studies in public health.

## List of abbreviations

ORFV: Orf virus; PCPV: pseudocowpox virus; BPSV: bovine papular stomatitis virus; SPPV: squirrel parapoxvirus; PVNZ: parapoxvirus of red deer in New Zealand; PCR: polymerace chain reaction; PBS: phosphate buffered solution; bp: base pair; kb: kilobase.

## Competing interests

The authors declare that they have no competing interests.

## Authors' contributions

XL was the leader of the project. KZ carried out most of the studies and drafted the manuscript. JH and JY amplified the complete B2L gene. HZ, ZL and YS provided consultation and preparation of the final report. All authors read and approved the final manuscript.

## References

[B1] Hosamani M Bhanuprakash V Scagliarini A Singh RK Comparative sequence analysis of major envelope protein gene (B2L) of Indian orf viruses isolated from sheep and goats Vet Microbiol 2006 116 317 324 10.1016/j.vetmic.2006.04.028 16777357

[B2] Inoshima Y Morooka A Sentsui H Detection and diagnosis of parapoxvirus by the polymerase chain reaction J Virol Methods 2000 84 201 208 10.1016/S0166-0934(99)00144-5 10680970

[B3] Pedersen AB Jones KE Nunn CL Altizer S Infectious diseases and extinction risk in wild mammals Conserv Biol 2007 21 1269 1279 10.1111/j.1523-1739.2007.00776.x 17883492 PMC7202242

[B4] Hubner G Loewe KR Dittmar FK [Human infection by the virus of contagious pustular dermatitis of sheep (author's transl)] Dtsch Med Wochenschr 1974 99 2392 2394 10.1055/s-0028-1108144 4473327

[B5] Paiba GA Thomas DR Morgan KL Bennett M Salmon RL Chalmers R Kench SM Coleman TJ Meadows D Morgan-Capner P Orf (contagious pustular dermatitis) in farmworkers: prevalence and risk factors in three areas of England Vet Rec 1999 145 7 11 10452390 10.1136/vr.145.1.7

[B6] Carr RW A case of orf (ecthyma contagiosum; contagious pustular dermatitis) contracted by a human from a wild Alaskan mountain goat Alaska Med 1968 10 75 77 4297665

[B7] Ara M Zaballos P Sanchez M Querol I Zubiri ML Simal E Horndler C Giant and recurrent orf virus infection in a renal transplant recipient treated with imiquimod J Am Acad Dermatol 2008 58 S39 40 10.1016/j.jaad.2006.04.027 18191701

[B8] Gallina L Dal Pozzo F Mc Innes CJ Cardeti G Guercio A Battilani M Ciulli S Scagliarini A A real time PCR assay for the detection and quantification of orf virus J Virol Methods 2006 134 140 145 10.1016/j.jviromet.2005.12.014 16430972

[B9] Vikoren T Lillehaug A Akerstedt J Bretten T Haugum M Tryland M A severe outbreak of contagious ecthyma (orf) in a free-ranging musk ox (Ovibos moschatus) population in Norway Vet Microbiol 2008 127 10 20 10.1016/j.vetmic.2007.07.029 17768017

[B10] Meynink SE Jackson PG Platt D Treatment of intraoral orf lesions in lambs using diathermy and cryosurgery Vet Rec 1987 121 594 3438994

[B11] McKeever DJ Jenkinson DM Hutchison G Reid HW Studies of the pathogenesis of orf virus infection in sheep J Comp Pathol 1988 99 317 328 10.1016/0021-9975(88)90052-7 3204166

[B12] Haig DM Mercer AA Ovine diseases. Orf Vet Res 1998 29 311 326 9689744

[B13] Robinson AJ Prevalence of contagious pustular dermatitis (orf) in six million lambs at slaughter: a three-year study N Z Vet J 1983 31 161 163 16030998 10.1080/00480169.1983.35008

[B14] Chan KW Lin JW Lee SH Liao CJ Tsai MC Hsu WL Wong ML Shih HC Identification and phylogenetic analysis of orf virus from goats in Taiwan Virus Genes 2007 35 705 712 10.1007/s11262-007-0144-6 17682935

[B15] Sullivan JT Mercer AA Fleming SB Robinson AJ Identification and characterization of an orf virus homologue of the vaccinia virus gene encoding the major envelope antigen p37K Virology 1994 202 968 973 10.1006/viro.1994.1420 8030257

[B16] Tikkanen MK McInnes CJ Mercer AA Buttner M Tuimala J Hirvela-Koski V Neuvonen E Huovilainen A Recent isolates of parapoxvirus of Finnish reindeer (Rangifer tarandus tarandus) are closely related to bovine pseudocowpox virus J Gen Virol 2004 85 1413 1418 10.1099/vir.0.79781-0 15166423

[B17] Inoshima Y Murakami K Yokoyama T Sentsui H Genetic heterogeneity among parapoxviruses isolated from sheep, cattle and Japanese serows (Capricornis crispus) J Gen Virol 2001 82 1215 1220 11297696 10.1099/0022-1317-82-5-1215

[B18] Guo J Rasmussen J Wunschmann A de La Concha-Bermejillo A Genetic characterization of orf viruses isolated from various ruminant species of a zoo Vet Microbiol 2004 99 81 92 10.1016/j.vetmic.2003.11.010 15019099

[B19] Guo J Zhang Z Edwards JF Ermel RW Taylor C Jr de la Concha-Bermejillo A Characterization of a North American orf virus isolated from a goat with persistent, proliferative dermatitis Virus Res 2003 93 169 179 10.1016/S0168-1702(03)00095-9 12782365

[B20] Pettersson B Uhlen M Johansson KE Phylogeny of some mycoplasmas from ruminants based on 16S rRNA sequences and definition of a new cluster within the hominis group Int J Syst Bacteriol 1996 46 1093 1098 10.1099/00207713-46-4-1093 8863441

[B21] Tryland M Klein J Nordoy ES Blix AS Isolation and partial characterization of a parapoxvirus isolated from a skin lesion of a Weddell seal Virus Res 2005 108 83 87 10.1016/j.virusres.2004.08.005 15681058

[B22] Thompson JD Higgins DG Gibson TJ CLUSTAL W: improving the sensitivity of progressive multiple sequence alignment through sequence weighting, position-specific gap penalties and weight matrix choice Nucleic Acids Res 1994 22 4673 4680 10.1093/nar/22.22.4673 7984417 PMC308517

[B23] Tamura K Dudley J Nei M Kumar S MEGA4: Molecular Evolutionary Genetics Analysis (MEGA) software version 4.0 Mol Biol Evol 2007 24 1596 1599 10.1093/molbev/msm092 17488738

[B24] Delhon G Tulman ER Afonso CL Lu Z de la Concha-Bermejillo A Lehmkuhl HD Piccone ME Kutish GF Rock DL Genomes of the parapoxviruses ORF virus and bovine papular stomatitis virus J Virol 2004 78 168 177 10.1128/JVI.78.1.168-177.2004 14671098 PMC303426

