# Diagnosis and phylogenetic analysis of Orf virus from goats in China: a case report

**DOI:** 10.1186/1743-422X-7-121

**Published:** 2010-06-09

**Authors:** Keshan Zhang, Youjun Shang, Ye Jin, Guangxiang Wang, Haixue Zheng, Jijun He, Zhongxin Lu, Xiangtao Liu

**Affiliations:** 1 Lanzhou Veterinary Research Institute of Chinese Academy of Agriculture Science, State KeyLaboratory of Veterinary Etiological Biology, National Foot-and-Mouth Disease ReferenceLaboratory, Key Laboratory of Animal Virology of Ministry of Agriculture, Xujiaping No.1, Yanchangpu, Lanzhou, Gansu, 730046, PR China

## Correction

After publication of this work [1], we noted that we inadvertently failed to include the complete list of all coauthors. The full list of authors has now been added and the Authors' contributions and Competing interests section modified accordingly.

## Competing interests

Financial support was provided by the National Modern Meat Caprine Industrial Technology System (nycytx-39), and the Integration and demonstration of foot-and-mouth disease comprehensive prevention and control of national technological support project (2006BAD06A17). Corresponding author is the unique person responsible for these projects.

All authors belongs to the same research unit(Lanzhou Veterinary Research Institute), so they have no conflicts of interest.

## Authors' contributions

KZ: carried out most of the studies and drafted the manuscript. YS: Study coordination and preparation of the final report. JY, JH: amplified the complete B2L gene. GW: Clinical investigation and sampling. HZ, ZL: consultation. XL: Idea, the leader of the project.

All authors read and approved the final manuscript.

## References

[B1] Zhang Keshan Lu Zhongxin Shang Youjun Zheng Haixue Jin Ye He Jijun Liu Xiangtao Diagnosis and phylogenetic analysis of Orf virus from goasts in China: a case report Virology Journal 2010 7 78 doi:10.1186/1743-422X-7-78 10.1186/1743-422X-7-78 20416112 PMC2877020

